# The Population Pharmacokinetic Models of Tacrolimus in Chinese Adult Liver Transplantation Patients

**DOI:** 10.1155/2014/713650

**Published:** 2014-02-13

**Authors:** Liqin Zhu, Hao Wang, Xiaoye Sun, Wei Rao, Wei Qu, Yuan Zhang, Liying Sun

**Affiliations:** ^1^Department of Pharmacy, Tianjin First Central Hospital, Tianjin 300192, China; ^2^Basic Medical College, Tianjin Medical University, Tianjin 300070, China; ^3^Department of Liver Transplantation, Tianjin First Central Hospital, Tianjin 300192, China; ^4^Department of Liver Transplantation, Beijing Friendship Hospital, Capital Medical University, Beijing 100050, China

## Abstract

*Aim*. The aim of this study was to establish population pharmacokinetic models of tacrolimus in Chinese adult liver transplantation patients. *Methods*. Tacrolimus dose and concentration data (*n* = 435) were obtained from 47 Chinese adult liver transplant recipients, and the data were analyzed using a nonlinear mixed-effect modeling (NONMEM) method. *Results*. The structural model was a two-compartment model with first-order absorption. The typical population values of tacrolimus for the pharmacokinetic parameters of apparent clearance (CL/*F*), apparent distribution volume of the central compartment (*V*_2_/*F*), intercompartmental clearance (*Q*/*F*), apparent distribution volume of the peripheral compartment (*V*_3_/*F*), and absorption rate (*k*_*a*_) were 11.2 L/h, 406 L, 57.3 L/h, 503 L, and 0.723 h^−1^, respectively. The interindividual variabilities of these parameters were 16.2%, 163%, 19.7%, 199%, and 74.3%, respectively, and the intraindividual variability of observed concentration was 26.54%. The covariates retained in the final models were postoperative days (POD) and dosage per day (DOSE) on CL/*F*. *Conclusion*. Population pharmacokinetic models of tacrolimus were developed in Chinese adult liver transplant patients. These results could provide the interpretation of the outcome of pharmacokinetics modeling and the impact of covariate tested on individualized tacrolimus therapy.

## 1. Introduction

Tacrolimus is a macrolide lactone immunosuppressant that is used clinically for the prophylaxis or reversal of organ rejection after organ transplantation. The administration of tacrolimus is complicated by a narrow therapeutic index, significant inter- and intrapatient variabilities in its pharmacokinetics, and toxicities [1]. Hence, therapeutic drug monitoring and dosage individualization based on patient pharmacokinetics are recommended when tacrolimus is used clinically to reduce the occurrence of adverse events and to optimize treatment outcomes. Currently, therapeutic drug monitoring is used to adjust the tacrolimus dosage and decrease the adverse effects [2, 3]. Though trough concentration monitoring is commonly used for dose individualization, the nephrotoxicity may exist when dosing is based on tacrolimus trough concentration alone. The area under the concentration-time curve is recognized as a measure for drug exposure [4, 5].

A population pharmacokinetic model can be used to predict the dosage regimen most likely to achieve a given target drug concentration based on patient characteristics (covariate values). A number of studies have been performed to characterize the pharmacokinetics of tacrolimus in liver transplant recipients. To date, many population pharmacokinetic studies both in adult and in pediatric liver transplant recipients [6–14] have been performed. From these previous studies, the patients' hepatic and renal function, body size, age (in pediatrics), time after transplant, and transplant type (whole or cutdown graft) were found to have influence on apparent clearance (CL/*F*) of tacrolimus; patients' size and haematocrit level were found to have influence on the apparent distribution volume (*V*/*F*) of tacrolimus.

However, the pharmacokinetic parameters observed in different races may not be applicable to Chinese adults liver transplant. There are limited data on the pharmacokinetics of tacrolimus in Chinese adults liver transplant patients. The primary aim of this study was to develop a population pharmacokinetic model to estimate the value of tacrolimus apparent clearance and to assess the intra- and interpatient variability in this parameter in a group of Chinese adult liver transplant recipients using both routine drug monitoring data and serial blood sample during the 12 h interval after administration of oral tacrolimus. The effects of various demographic, hematological, and biochemical parameters on tacrolimus disposition were also investigated.

## 2. Method

### 2.1. Patients and Data Collection

Data were collected prospectively from Chinese adult liver transplant patients who had been administered tacrolimus at the Tianjin First Hospital, China, from 2008 to 2011. Approval was obtained from the hospital's ethics committee for the study. Informed verbal consent was obtained from the patients or their caregivers for blood sampling in addition to those required for routine therapeutic drug monitoring.

Patient demographic characteristics including age, gender, dosage per day (DOSE), and postoperative days (POD) were collected in hospital medical records system. In addition, the data on biochemical and hematological indices including alanine aminotransferase (ALT), aspartate aminotransferase (AST), alkaline phosphatase (ALP), total bilirubin (TBIL), serum creatinine (SCr), haematocrit (Hct), white blood cell (WBC), and red blood cell were also collected. All data were collected only from the inpatients.

The patients' weight and transplant type (whole or cut) were not collected because of so much missing information from the inpatients.

### 2.2. Drug Administration

All patients received oral tacrolimus (capsules, 1 mg and 0.5 mg) therapy as part of triple immunosuppressive regimen, which also included mycophenolate mofetil and corticosteroids. Therapy was generally initiated at a dosage between 0.1 and 0.15 mg/kg twice daily. Subsequent doses were adjusted empirically on the basis of clinical evidence of efficacy and toxicity and to maintain tacrolimus trough blood concentrations between 10 and 15 ng/mL in the first 3 months after transplant. In the immediate posttransplantation (intensive care unit) period, blood samples were collected daily (before the morning dose) until concentrations were stabilized. Then, blood samples from inpatients were collected three times weekly or more frequently if justified (suspicion of rejection or adverse event). In our study, all blood concentrations were obtained in steady-state conditions. Most of them were collected before the dose and at 0.3, 1, 1.5, 2, 4, 6, 8, and 12 hours after dose, while some were collected only before administration.

### 2.3. Analytical Method

Concentrations of tacrolimus in whole blood were assessed using MEIA (microparticle enzyme immunoassay) performed on the IMx platform. According to manufacturer's information, the lower limit of quantification of the assay was 1.5 *μ*g/L, and it was linear over the range 1.5–30 ng/mL. Blood samples exceeding the upper limit of the calibration range (30 ng/mL) were diluted according to the manufacturer's protocol. Although the antitacrolimus monoclonal antibody recognizes not only the parent drug but also three of its metabolites (M-II, M-III, and MV), the cross-reactivity for other metabolites was less than the minimum detectable sensitivity. The values of interassay variability (coefficient of variation, CV%) with tacrolimus concentrations of 5, 11, and 22 ng/mL were 1.7, 1.8, and 2.8%, respectively. The values of intra-assay variability (CV%) with 5, 11, and 22 ng/mL were 8.7, 5, and 4.1%, respectively (manufacturer's information).

### 2.4. Population Pharmacokinetic Modeling

Pharmacokinetic analysis was carried out using the nonlinear, mixed-effects modeling program NONMEM (version V; level 1.1; GloboMax LLC, Hanover, MD, USA). The population analysis was undertaken using the first-order conditional estimation (FOCE) method with interaction. One- and two-compartment pharmacokinetic models were compared. The structural models were chosen according to the objective function values (OFV) and the goodness of fit of the models. The bioavailability (*F*) and absorption with a lag time could not be determined because tacrolimus was orally administered and the blood data included many sparse blood data; therefore, the pharmacokinetic values of clearance (CL), distribution volume (*V*), and intercompartmental clearance (*Q*) corresponded to the ratios of CL/*F* (apparent clearance), *V*/*F* (apparent distribution volume), and *Q*/*F* (intercompartmental clearance), respectively.

### 2.5. Random Effect Model

Interindividual variability models of the tacrolimus pharmacokinetic parameters were evaluated using additive, proportional, and log normal models. The residual error model was also tested using an additive, proportional, and combined (proportion plus additive) model.

### 2.6. Covariate Model

The selection between models was based on the precision of parameter estimates, goodness of fit, and the minimum value of the NONMEM objective function value [−2 log (likelihood)].

Covariates were initially screened using scatter plots of individual pharmacokinetics and by generalized additive modeling using SAS 9.1.

The potential covariates that were screened by generalized additive modeling were individually introduced into the basic model and screened again with NONMEM. The effect of a covariate was assessed by *χ* ^2^ testing of the difference between the OFV of the basic model and the incorporated covariate model. A decrease in ΔOFV > 3.84 (degree of freedom, d.f. = 1) when a covariate was incorporated was considered significant at *α* = 0.05; potential significant covariates were also screened.

Next, the potential significant covariates according to the size of ΔOFV, from large to small, were incorporated in order using forward inclusion. A decrease in ΔOFV > 6.64 (d.f. = 1) when a potential significant covariate was incorporated was considered significant at *α* = 0.01. A full regression model was established in this way.

The final regression model was then established by stepwise backward elimination from the full regression model. A change of ΔOFV > 7.88 (d.f. = 1) was considered significant at *α* = 0.005 when a covariate was removed.

A number of covariates from Table 1, including demographic characteristics and hematological and biological indices, were analyzed using forward included and stepwise backward elimination. The covariates were divided into continuous and dichotomous variables. Additive, exponential, and power models were tested for continuous variables, while power models were evaluated for dichotomous variables.

### 2.7. Evaluation of Population Pharmacokinetic Parameters

The accuracy and robustness of the final model were simultaneously evaluated using the resampling techniques of bootstrap and visual predictive check (VPC).

Bootstrap is a resampling technique that gets a data set from the original data by N random draws [15, 16]. This process was performed using the software package Wings for NONMEM and repeated 500 times with different random draws. Bootstrap results for which the minimization was successful and covariance was acceptable were used for further analysis. The medians and 2.5–97.5% percentiles of the bootstrap data set parameters were compared to the final pharmacokinetic parameter estimates.

VPC is a valuable method for checking model performance. It is used to graphically evaluate an established model [17]. The rationale of VPC is to simulate a new data set according to the final model parameters and then fit the new data set with the final model and determine the parameters for the new data set. Plotting of the observed concentrations and 90% prediction intervals of simulated concentrations versus time was performed with the assistance of R for NONMEM and Wing for NONMEM.

## 3. Results

### 3.1. Patients and Data Collection

Data were collected prospectively from 47 Chinese adult liver transplant recipients during hospitalization. The mean duration of hospitalization was 20 ± 18 days (range 2–85). A total of 435 whole blood tacrolimus concentrations were collected from 47 Chinese adult liver transplant patients. The other sparse samples were collected at the end of the dosing interval. Patient characteristics are listed in Table 1.

### 3.2. Population Pharmacokinetic Model Analysis

A two-compartment model with first-order absorption was better fitted to the data than one-compartment model, as noted by a greater reduction of the objective function value of 28.38 (*P* < 0.001). The pharmacokinetic parameters of tacrolimus that were estimated by NONMEM included CL/*F*, apparent distribution volume of the central compartment (*V*_2_/*F*), *Q*/*F*, apparent distribution volume of the peripheral compartment (*V*_3_/*F*), and absorption rate. The interindividual variability model was ultimately evaluated using a log normal model, as shown in

(1) $$\begin{array}{c} \theta_{ij}=\theta \times \exp\left(\eta_{ij}\right), \end{array}$$

where *θ*_*ij*_ represents the *i*th individual value of the parameter on the *j*th occasion, *θ* represents the typical population value of the parameter, *η* represents the interindividual variability of the pharmacokinetic parameter, and *η* is a symmetric distribution with a zero mean and variance of *ω* ^2^.

The residual error model was evaluated using the proportional error model, which is equal to the log error model. The residual error model is shown in

(2) $$\begin{array}{c} Y=\text{IPRED}\times \left(1+\varepsilon \right), \end{array}$$

where *Y* represents the observed concentration, IPRED represents the individual predicted concentration, which was simulated by POSTHOC with NONMEM, and *ε* is the residual error, which was randomly distributed with a zero mean and variance of *σ* ^2^. The basic model pharmacokinetic parameter estimates are given in Table 2.

Eleven covariates were analyzed in the present study, and only the following covariates showed significant influence on the pharmacokinetic parameters: dose and postoperative days on CL/*F*. The changes of the objective function values are shown in Table 3.

The final covariate models were as follows:

(3) $$\begin{array}{c} \frac{\text{CL}}{F}=\theta_{\text{CL}/F}\times {\text{DOSE}}^{\theta \text{DOSE}}\times {\text{POD}}^{\theta \text{POD}}, \end{array}$$

where *θ*_CL/*F*_ are the typical population values of CL/*F* and *θ*_DOSE_ and *θ*_POD_ are the coefficients of the dose and pod, respectively.

**Figure 1:**
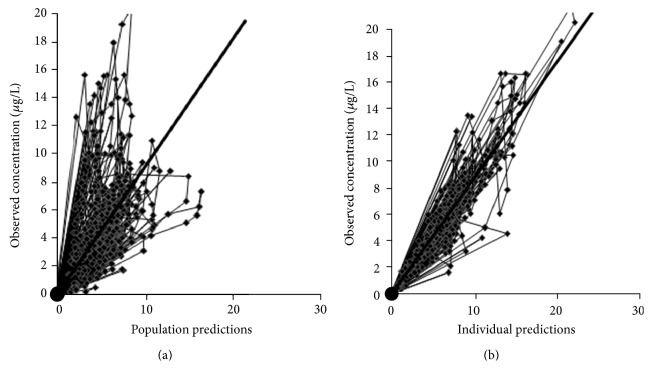
Fit plots of the final model: observations versus population predictions (a) and observations versus individual predictions (b). The bold solid lines represent the lines of unity.

The goodness of fit of the final model is shown in Figure 1. The plot (a) showed observations versus population predictions and plot (b) showed observations versus individual predictions. The two plots, respectively, showed good individual and population predicted results.

### 3.3. Model Validation

**Figure 2:**
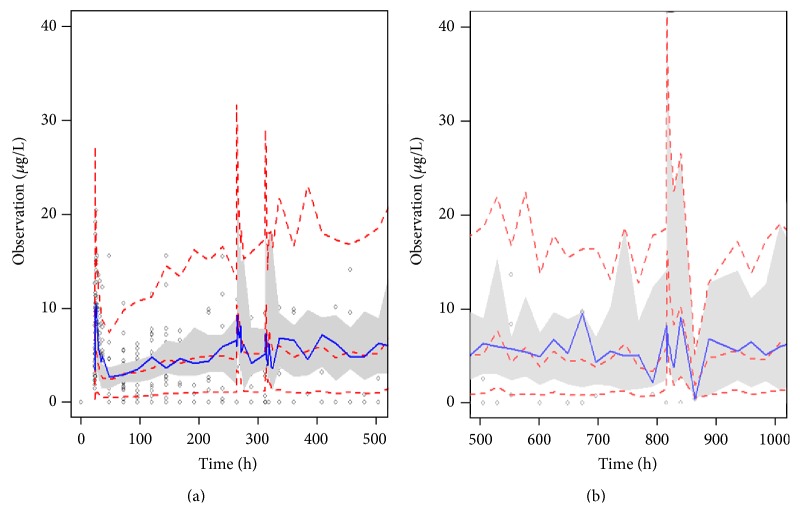
VPC profile of the final model. The plot (a) is the prediction to observed concentrations in first month after transplantation. Plot (b) is that after the first month of transplantation. The middle solid line represents the median of the simulated data. The lower and upper dashed lines are the 5th and 95th percentiles of the simulated data, respectively.

Model validation is very important in population analyses. In the present study, bootstrap and VPC were used to evaluate model stability. The results of the bootstrap method are shown in Table 4. The distribution of the simulated concentrations and observed concentrations versus time is shown in Figure 2.

### 3.4. Final Pharmacokinetic Parameter Estimates

The results of the final model pharmacokinetic parameters are presented in Table 4.

## 4. Discussion

In this study, the pharmacokinetics of tacrolimus was investigated in Chinese adult liver transplant patients by a population modeling approach. Our results indicated that the DOSE and POD significantly affect CL/*F*. The interindividual variability of the final model was decreased compared with the parameters in the basic model. The final model had goodness of fit to the data set in the present study.

POD was identified as a major covariate that described the recovery of tacrolimus CL. This covariate has been already identified in liver transplant recipients, renal transplant recipients, and paediatric transplant recipients [18, 19]. Aweeka et al. [20] reported that the clearance of tacrolimus was higher in post- than pre-kidney transplants recipients. Several studies in adults have reported an increase in the dose of tacrolimus required to maintain similar trough concentrations with increasing time after transplantation [21].

Indices of renal function (serum creatinine), gender, haematocrit, and albumin had no significant effect on the CL of tacrolimus. The lack of effect of indices of renal function on CL is plausible since the renal clearance of tacrolimus accounts for less than 1% of total systemic clearance. Tacrolimus is a low-clearance drug; the extraction ratio is equivalent to about 3% of liver blood flow. For a highly bound, lower-extraction ratio drug like tacrolimus, clearance would be affected by changes in haematocrit and plasma protein binding. Indeed, trough whole-blood concentration of tacrolimus after renal transplantation correlates with haematocrit and albumin during the first weeks of treatment and its relative clearance was negatively correlated with haematocrit and albumin. However, the lack of significant effect of haematocrit and albumin on CL in the current study could be attributed to the great range of haematocrit and albumin concentrations and not too large samples in this study. The increasing of sample should be one of the directions of improvement of this paper.

The typical value of the absorption rate (0.723 h^−1^) was lower than that in patients [22, 23] whose absorption rates were all fixed in 4.5 h^−1^. This could be due to the P-glycoprotein function. P-glycoprotein, which is encoded by ABCB1, acts as a barrier for drugs such as tacrolimus because of its efflux pump activity in the intestine [24, 25]. The genetic variations of ABCB1 SNPs may be associated with altered P-gp function. Also this could be simply due to the race difference between the western and eastern population. This could be approved in another paper [25]. Because of seldom comedication with other drugs that influence the concentration of tacrolimus, such as fluconazole and diltiazem [26], our present study was not designed to investigate drug-drug interactions.

The final models were validated using bootstrap and VPC. The result of bootstrap showed that the median values of the parameters were close to the final model estimates. The distribution of the simulated concentration-time curves was compared with the observed concentrations. The final models accurately predicted the tacrolimus pharmacokinetic process in Chinese adult liver transplantation patients.

## 5. Conclusion

The population pharmacokinetics of tacrolimus was studied in 47 Chinese liver transplant patients in the present study, and population pharmacokinetic models were developed. The following covariates were retained in the final model: DOSE and POD on CL/*F*. Our results provide a reference for individualized tacrolimus therapy in the clinical setting.

## Figures and Tables

**Table 1:** Characteristics of the 47 adult patients (27 male/20 female).

Characteristics	Mean	SD	Median	Range
Gender (male/female)	(27/20)	—	—	—

Age (y)	57.47	11.16	60	25–78
Dose (mg/d)	5.31	2.27	5	1–10.5
Postoperative days (d)	20.71	18.04	14	2–85
Alkaline phosphatase (IU/L)	108.1	61.22	98.3	23.5–385.6
Alanine amino transferase (IU/L)	105.8	99.1	72.2	9.8–72.2
Aspirate amino transferase (IU/L)	41.38	39.62	29.9	9.6–332.1
Total bilirubin (*μ*mol/L)	47.02	60.55	27.47	2.82–436.7
Serum creatinine (*μ*mol/L)	92.95	80.37	67.5	25.9–520.3
Haemotocrit (%)	28.94	5.7	28	2–83.4
White blood cell (∗10^9^/L)	7.85	3.85	7.32	1.52–41.1
Red blood cell (∗10^12^/L)	3.23	0.73	3.07	2–8.99

**Table 2:** Parameter estimates of the basic model.

Parameter	Estimate	SE%
CL/*F* (L/h)	30.2	0.248
*V*_2_/*F* (L)	294	8.79
*Q*/*F* (L/h)	78.1	1.54
*V*_3_/*F* (L)	327	7.98
*k*_*a*_ (h^−1^)	0.473	22.9
*ω*CL/*F* (%)	22.6	6.19
*ωV*_2_/*F* (%)	150	39.8
*ωQ*/*F* (%)	10.6	5.87
*ωV*_3_/*F* (%)	145	43.8
*ωk*_*a*_ (%)	52.9	30.8
*σ* (%)	8.31	1.06

**Table 3:** Change of objective function value of covariate analysis.

	OFV	ΔOFV	*P* value
Inclusion			
Basic model	1008.606	—	—
Influence of DOSE on CL	957.592	−51.014	<0.05
Influence of POD on CL	936.480	−21.112	<0.05
Influence of DOSE on *V*_2_	931.680	−4.8	<0.05
Elimination			
Full model	931.680	—	—
Eliminate DOSE on *V*_2_	936.480	4.8	>0.01
Eliminate POD	955.597	23.917	<0.01
Eliminate DOSE on CL	941.752	10.072	<0.01

**Table 4:** Parameter estimates of final model and bootstrap validation.

Parameter typical	Parameter estimate	SE%	Bootstrap	
			Median	2.5th, 97.5th percentiles
CL/*F* (L/h)	11.2	0.28	11.7	7.59, 18.4
*V*_2_ (L)	406	30	375	262, 570
*Q* (L/h)	57.3	4.86	66.3	48.5, 113
*V*_3_ (L)	503	18.5	526	363, 853
*k*_*a*_ (h^−1^)	0.723	72.4	0.764	0.545, 1.43
*θ*DOSE	0.371	11.1	0.337	0.14, 0.511
*θ*POD	0.127	3.8	0.127	0.0906, 0.183
*ω*CL/*F* (%)	16.2	4.67	23.98	10.82, 40.2
*ωV*_2_/*F* (%)	163	164	120	67.2, 170
*ωQ*/*F* (%)	19.7	21.4	26.5	8.9, 47.8
*ωV*_3_/*F* (%)	199	231	127	79.7, 237
*ωk*_*a*_ (%)	74.3	57.5	81.91	40.2, 110
*σ* (%)	26.54	0.868	25.1	22.1, 28.2

## References

[1] Staatz C. E., Tett S. E. (2004). Clinical pharmacokinetics and pharmacodynamics of tacrolimus in solid organ transplantation. *Clinical Pharmacokinetics*.

[2] Wallin J. E., Friberg L. E., Fasth A., Staatz C. E. (2009). Population pharmacokinetics of tacrolimus in pediatric hematopoietic stem cell transplant recipients: new initial dosage suggestions and a model-based dosage adjustment tool. *Therapeutic Drug Monitoring*.

[3] Musuamba F. T., Mourad M., Haufroid V., Delattre I. K., Verbeeck R. K., Wallemacq P. (2009). Time of drug administration, CYP3A5 and ABCB1 genotypes, and analytical method influence tacrolimus pharmacokinetics: a population pharmacokinetic study. *Therapeutic Drug Monitoring*.

[4] Mathew B. S., Fleming D. H., Jeyaseelan V. (2008). A limited sampling strategy for tacrolimus in renal transplant patients. *British Journal of Clinical Pharmacology*.

[5] Miura M., Satoh S., Niioka T. (2009). Early phase limited sampling strategy characterizing tacrolimus and mycophenolic acid pharmacokinetics adapted to the maintenance phase of renal transplant patients. *Therapeutic Drug Monitoring*.

[6] Antignac M., Hulot J. S., Boleslawski E. (2005). Population pharmacokinetics of tacrolimus in full liver transplant patients: modelling of the post-operative clearance. *European Journal of Clinical Pharmacology*.

[7] Fukatsu S., Yano I., Igarashi T. (2001). Population pharmacokinetics of tacrolimus in adult recipients receiving living-donor liver transplantation. *European Journal of Clinical Pharmacology*.

[8] Macchi-Andanson M., Charpiat B., Jelliffe R. W., Ducerf C., Fourcade N., Baulieux J. (2001). Failure of traditional trough levels to predict tacrolimus concentrations. *Therapeutic Drug Monitoring*.

[9] Wai J. S., Lai S. T., Holmes M. J. (2006). Population pharmacokinetics of tacrolimus in whole blood and plasma in Asian liver transplant patients. *Clinical Pharmacokinetics*.

[10] Staatz C. E., Willis C., Taylor P. J., Lynch S. V., Tett S. E. (2003). Toward better outcomes with tacrolimus therapy: population pharmacokinetics and individualized dosage prediction in adult liver transplantation. *Liver Transplantation*.

[11] Fukudo M., Yano I., Masuda S. (2006). Population pharmacokinetic and pharmacogenomic analysis of tacrolimus in pediatric living-donor liver transplant recipients. *Clinical Pharmacology and Therapeutics*.

[12] Garcia Sanchez M. J., Manzanares C., Santos-Buelga D. (2001). Covariate effects on the apparent clearance of tacrolimus in paediatric liver transplant patients undergoing conversion therapy. *Clinical Pharmacokinetics*.

[13] Staatz C. E., Taylor P. J., Lynch S. V., Willis C., Charles B. G., Tett S. E. (2001). Population pharmacokinetics of tacrolimus in children who receive cut-down or full liver transplants. *Transplantation*.

[14] Wallin J. E., Bergstrand M., Wilczek H., Nydert P. S., Karlsson M. O., Staatz C. E. (2011). Population pharmacokinetics of tacrolimus in pediatric liver transplantation: early posttransplantation clearance. *Therapeutic Drug Monitoring*.

[15] Brendel K., Dartois C., Comets E. (2007). Are population pharmacokinetic and/or pharmacodynamic models adequately evaluated? A survey of the literature from 2002 to 2004. *Clinical Pharmacokinetics*.

[16] Jolling K., Perez Ruixo J. J., Hemeryck A., Vermeulen A., Greway T. (2005). Mixed-effects modelling of the interspecies pharmacokinetic scaling of pegylated human erythropoietin. *European Journal of Pharmaceutical Sciences*.

[17] Post T. M., Freijer J. I., Ploeger B. A., Danhof M. (2008). Extensions to the Visual Predictive Check to facilitate model performance evaluation. *Journal of Pharmacokinetics and Pharmacodynamics*.

[18] Antignac M., Hulot J. S., Boleslawski E. (2005). Population pharmacokinetics of tacrolimus in full liver transplant patients: modelling of the post-operative clearance. *European Journal of Clinical Pharmacology*.

[19] Staatz C. E., Willis C., Taylor P. J., Lynch S. V., Tett S. E. (2003). Toward better outcomes with tacrolimus therapy: population pharmacokinetics and individualized dosage prediction in adult liver transplantation. *Liver Transplantation*.

[20] Aweeka F. T., Benet L. Z., Gambertoglio J. G. (1993). Comparative pharmacokinetics of orally (PO) and intravenously (IV) administered tacrolimus (FK506) in pre- and post-kidney transplant recipients. *Clinical Pharmacology & Therapeutics*.

[21] Undre N. A., Schäfer A. (1998). Factors affecting the pharmacokinetics of tacrolimus in the first year after renal transplantation. *Transplantation Proceedings*.

[22] Press R. R., Ploeger B. A., Hartigh J. D. (2009). Explaining variability in tacrolimus pharmacokinetics to optimize early exposure in adult kidney transplant recipients. *Therapeutic Drug Monitoring*.

[23] Staatz C. E., Goodman L. K., Tett S. E. (2010). Effect of CYP3A and ABCB1 single nucleotide polymorphisms on the pharmacokinetics and pharmacodynamics of calcineurin inhibitors: part I. *Clinical Pharmacokinetics*.

[24] Marzolini C., Paus E., Buclin T., Kim R. B. (2004). Polymorphisms in human MDR1 (P-glycoprotein): recent advances and clinical relevance. *Clinical Pharmacology and Therapeutics*.

[25] Shi X.-J., Geng F., Jiao Z., Cui X.-Y., Qiu X.-Y., Zhong M.-K. (2011). Association of ABCB1, CYP3A4∗18B and CYP3A5∗3 genotypes with the pharmacokinetics of tacrolimus in healthy Chinese subjects: a population pharmacokinetic analysis. *Journal of Clinical Pharmacy and Therapeutics*.

[26] Zahir H., McLachlan A. J., Nelson A., McCaughan G., Gleeson M., Akhlaghi F. (2005). Population pharmacokinetic estimation of tacrolimus apparent clearance in adult liver transplant recipients. *Therapeutic Drug Monitoring*.

